# Restoration of horizontal stability in complete acromioclavicular joint separations: surgical technique and preliminary results

**DOI:** 10.1186/2047-783X-18-42

**Published:** 2013-11-13

**Authors:** Haoqing Li, Chuanshun Wang, Jiandong Wang, Kai Wu, Donghua Hang

**Affiliations:** 1 Department of Orthopedic Surgery, Shanghai First People’s Hospital Affiliated to Shanghai Jiaotong University, 100 Haining Road, Hongkou District, Shanghai 200080, China

**Keywords:** Acromioclavicular joint dislocation, Acromioclavicular ligament, Arthroscope-assisted, Horizontal stability, Reconstruction

## Abstract

**Background:**

Our purpose was to investigate the clinical efficacy of arthroscope-assisted acromioclavicular ligament reconstruction in combination with double endobutton coracoclavicular ligament reconstruction for the treatment of complete acromioclavicular joint dislocation.

**Methods:**

During the period from February 2010 to October 2012, ten patients with Rockwood types IV and V acromioclavicular joint dislocation were hospitalized and nine were treated with acromioclavicular ligament reconstruction combined with double endobutton of coracoclavicular ligament reconstruction. The improvement in shoulder functions was assessed using a Constant score and visual analog scale (VAS) system.

**Results:**

The mean follow-up period was 33.6 ± 5.4 months. The mean Constant scores improved from 25.2 ± 6.6 preoperatively to 92.4 ± 6.5 postoperatively, while the mean VAS score decreased from 5.9 ± 1.4 to 1.2 ± 0.9; significant differences were observed. The final follow-up revealed that excellent outcomes were achieved in eight patients and good outcome in two patients.

**Conclusion:**

Arthroscope-assisted acromioclavicular ligament reconstruction in combination with double endobutton of coracoclavicular ligament reconstruction is an effective approach for treatment of acute complete acromioclavicular joint dislocation.

## Background

Acromioclavicular joint dislocation is a common injury, which accounts for about 9% of all shoulder injuries [1]. It ranks second only to glenohumeral joint dislocation, with a higher incidence in athletes.

The biomechanics of the acromioclavicular joint involve static stability, dynamic stability, and acromioclavicular joint motion. Static stability is supplied by the acromioclavicular, coracoclavicular, and coracoacromial ligaments, while dynamic stability is maintained by the deltoid and trapezius muscles [2]. According to the Rockwood classification [3], both static instability and dynamic instability occur in types IV to VI. Therefore, surgical treatment is advised for treatment of Rockwood types IV to VI, while conservative treatment is employed for treatment of Rockwood types I and II [1,4]. However, there is a discrepancy in the treatment of Rockwood type III [5].

Since Cooper firstly introduced the treatment of surgical fixation in 1861, surgical techniques for reconstructing or repairing acromioclavicular joint dislocation have evolved over the last decades [6]. However, a golden standard procedure has not emerged. The majority of previous procedures focus on reconstruction of the coracoclavicular ligament to recover the stability of acromioclavicular joint, but few studies are reported regarding acromioclavicular ligament reconstruction. Moreover, biomechanical studies demonstrate that the horizontal stability of the acromioclavicular ligament cannot be completely restored by reconstruction of other ligaments [7].

A double endobutton technique was first introduced by Struhl [8]. In his study, he modified an endobutton coracoclavicular ligament device by adding a second endobutton to the construct, creating a knotless fixation. In this study, we combined arthroscope-assisted acromioclavicular ligament reconstruction with this double endobutton technique of coracoclavicular ligament reconstruction, to treat acute complete acromioclavicular joint dislocation, so as to investigate the role of acromioclavicular ligament reconstruction in treatment of acromioclavicular joint dislocation.

## Methods

### Subjects

All studies conformed to the World Medical Association Declaration of Helsinki (June 1964) and subsequent amendments. The research protocol was approved by the local ethical committee or equivalent (Shanghai First People’s Hospital Affiliated to Shanghai Jiaotong University ). In total, ten patients with acromioclavicular joint dislocation receiving acromioclavicular ligament reconstruction during the period from February 2010 to October 2012 were enrolled, including five men and five women, with an average age of 46.4 ± 13.1 years old. The dominant shoulder was involved in five patients (50%). The causes were traffic accident injury in five cases, falling injury in four cases and sports injury in one case, and all were closed injuries. According to the Rockwood classification [3], type IV dislocation (the clavicle is displaced posteriorly into the trapezius muscle) was found in seven cases, and type V dislocation (the clavicle is elevated between 100% and 300%) was observed in three cases. One case was complicated by craniocerebral trauma, four cases were complicated by multiple rib fractures, two cases were complicated by pneumothorax, and three cases were complicated by scapular fractures. The average time from injury to surgery was 4.3 ± 2.7 days.

Diagnosis of acromioclavicular joint dislocation relied on physical examination and radiographic inspection. Clinical signs included swelling of the injured shoulder, tenderness of the acromioclavicular joint, a positive piano-key sign, a bone rubbing feeling and limitation of shrug activity. The horizontal stability of the acromioclavicular joint was assessed. For this evaluation, the clinician stabilized the acromion of the normal shoulder with one hand, grasped the midshaft of the affected clavicle with the other hand, and gently translated the affected clavicle anterior-posteriorly. Radiographic evaluations included anteroposterior X-ray of the bilateral acromioclavicular joint, Zanca view oblique X-ray, and an additional stress X-ray for the dislocation with unknown classification. If Rockwood type IV injury with horizontal instability was suspected during the examination, computerized tomography (CT) and three-dimensional imaging were performed to identify the posterior translation of the distal clavicle.

### Surgical procedure

All patients underwent general anesthesia. The patient lay in a beach-chair position with the head turned away from the injured shoulder. A vertical incision was made from the posterior edge of the acromioclavicular joint toward the coracoid tip along the Langer’s line. Medial and lateral skin flaps were slightly developed. Any rupture of the deltoid and trapezius fascia was visualized and these were reattached if necessary. The anterior part of the deltoid was split along with its fibers, and the coracoid process was visualized and cleared off all the way to the base. The medial and lateral edges of the coracoids at the base and the knee were carefully identified.

**Figure 1:**
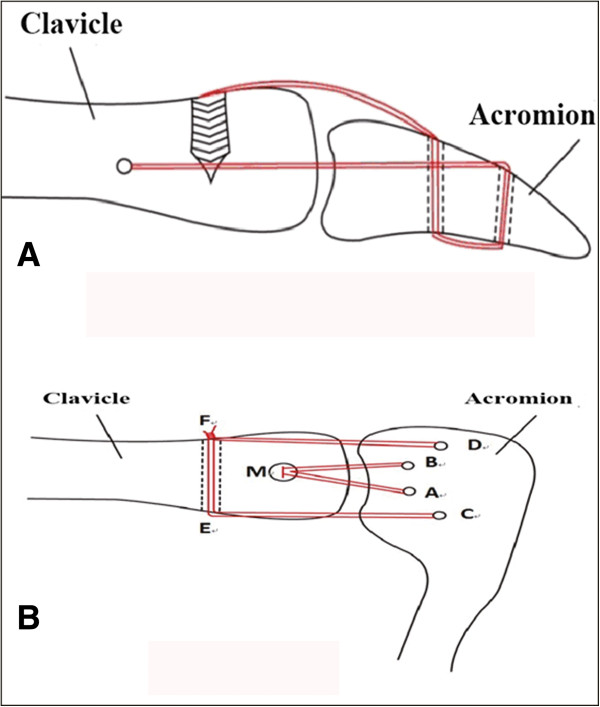
**Diagram for acromioclavicular ligament reconstruction. (A)** Anteroposterior view; **(B)** vertical view.

Reconstruction of the coracoclavicular ligament was performed using the double endobutton technique introduced by Struhl [8]. The operation steps of reconstruction of the acromioclaviclar ligament are as follows: Firstly, the shoulder arthroscope was inserted into the subacromial space from the posterior portal, which was 1 cm inferior and medial to the posterior corner of the acromion, and the lateral portal was selected as the performing passage. A suture anchor (M in Figure 1) (Mitek FASTIN RC, Depuy) was screwed into the distal clavicle 0.5 cm from the acromioclavicular joint (Figure 1), and two bone holes were drilled in the acromion at 0.5 cm from the acromioclavicular joint with an interval of 0.8 cm (A and B in Figure 1). In addition, two bone holes were drilled in the acromion at 0.8 cm from the acromioclavicular joint with an interval of 2 cm, namely in the site approximate to the extending line of the anterior and posterior edges of the clavicle (C and D in Figure 1). The four strands of sutures were divided into two groups. One group of sutures was threaded into the subacromial space via hole A from top to bottom, and a polydioxanone (PDS) thread was threaded into the subacromial space via hole C from top to bottom. Under arthroscopy, the tails of the sutures and PDS thread were pulled out from the lateral performing passage and knotted together. Then the sutures were pulled through hole C from bottom to top by pulling the PDS end upon the acromion. In the same way, the other group of sutures was threaded downward through hole B, and then crossed upward through hole D. Then a bone hole was drilled in the distal clavicle 0.8 cm from the acromioclavicular joint from anterior to posterior (E and F in Figure 1), and this group of sutures was threaded through this bone hole and knotted with the first group of sutures. Before knotting, the acromioclavicular joint dislocation should be completely reduced, and the two groups of sutures should be tightened. Subsequently, the sutures MA and MB functioned as a reconstruction of the superior strut of the acromioclavicular ligament, while the sutures CE and DF functioned as a reconstruction of the anterior and posterior struts, respectively. The deltoid and trapezius fascia were sutured before the wound was closed.

### Postoperative management and evaluation

Postoperatively, passive motion of the shoulder was carried out under the guidance of physiotherapists. Four weeks later, progressive active exercise of the shoulder was started, and daily activities resumed 3 months after surgery, while sporting activities were permitted after 6 months of surgery.

Each patient was followed up every 4 weeks for 6 months, every 3 months for another 18 months, and annually thereafter. The follow-up included clinical inquiry and physical examination, and X-rays were taken. A Constant shoulder score and visual analog scale (VAS) system was employed for outcome assessment in the final follow-up. According to the grading of the Constant shoulder score [9], an excellent outcome was defined as a score between 90 and 100 and a good outcome was defined as a score between 75 and 90.

### Statistical analysis

All statistical analyses were performed using the Statistical Package for Social Sciences (SPSS) version 13.0 (SPSS Inc., USA). The differences in the Constant and VAS scores were tested for statistical significance with the paired *t* test before and after surgery, with a *P* value <0.05 considered statistically significant.

## Results

**Figure 2:**
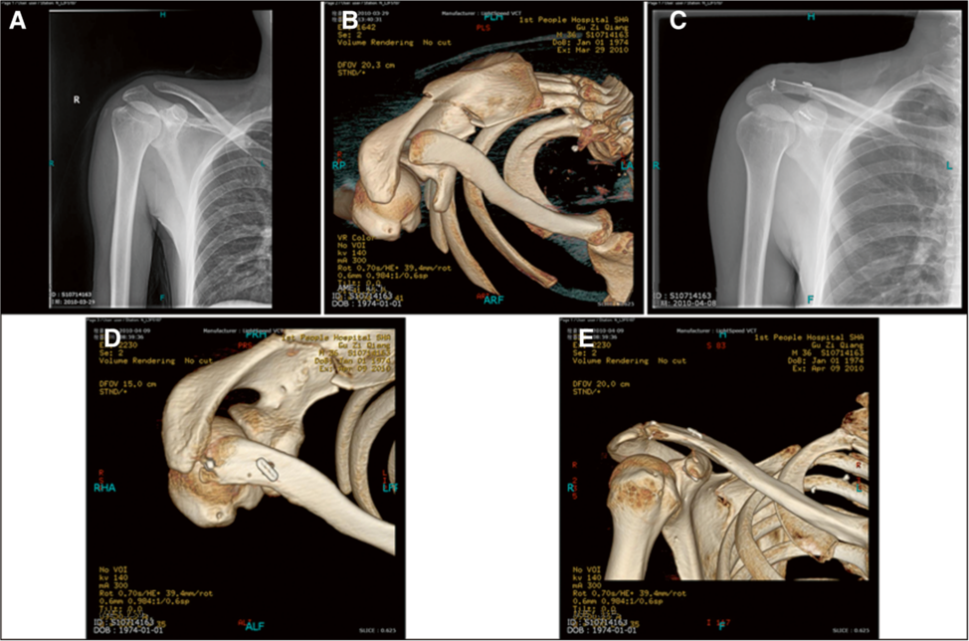
**Treatment of Rockwood type IV dislocation. (A)** Preoperative anteroposterior X-ray shows acromioclavicular joint space widening. **(B)** Preoperative CT scan demonstrates that the distal clavicle is displaced backwards, indicating Rockwood type IV dislocation. **(C–E)** Postoperative X-ray and CT scan indicate that both the vertical and horizontal positions achieve the anatomic reduction.

**Figure 3:**
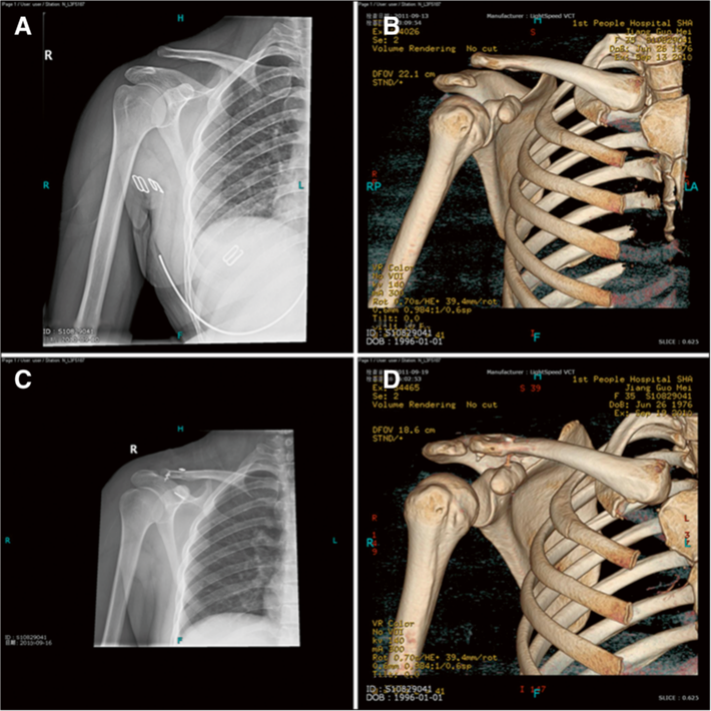
**Treatment of Rockwood type V dislocation. (A,B)** Preoperative X-ray and CT scan indicate Rockwood type V acromioclavicular joint dislocation. **(C,D)** Postoperative X-ray and CT scan reveal complete reduction of the dislocation.

Of the total ten cases, seven patients with Rockwood type IV dislocation and three patients with Rockwood type V dislocation underwent surgical treatment of arthroscope-assisted acromioclavicular ligament reconstruction in combination with a double endobutton technique for coracoclavicular ligament reconstruction (Figures 2 and 3). One patient (case 2) with acromioclavicular joint dislocation complicated by acromial and coracoid fractures with Rockwood type IV dislocation, with an entire coracoclavicular ligament, did not receive the double endobutton treatment for coracoclavicular ligament reconstruction, but only underwent acromioclavicular ligament reconstruction to stabilize the acromioclavicular joint, tension band fixation for the acromial fracture and screw fixation for the coracoid fracture. The four weeks follow-up after surgery found mild upward translation on the distal clavicle in comparison with that on the opposite side, and no further aggravation of the translation was observed thereafter. The final Constant score was 83, and gross satisfactory function was achieved.

All ten subjects were followed up (Table 1), with a mean follow-up period of 33.6 (24 to 40) months. During the follow-up period, no complications of infections, clavicle or coracoid fracture, or endobutton translation were observed. According to the grading of the follow-up Constant scores, excellent therapeutic efficacy was achieved in eight cases (80%), and good therapeutic efficacy was achieved in two cases (20%) (Table 2). The Constant shoulder scores were significantly improved after surgery (25.2 ± 6.6, preoperatively vs. 92.4 ± 6.5, postoperatively; *P* < 0.001) (Table 2). The mean VAS score was 1.2 ± 0.92 after surgery, compared with 5.9 ± 1.5 before surgery, and a significant difference was found (*P* < 0.001) (Table 3).

**Table 1:** Demographic characteristics of the patients

**Case**	**Sex**	**Age (years)**	**Injury side**	**Rockwood classification**	**Time from injury to surgery (days)**	**Combined injury**
1	Male	36	Right	IV	11	Multiple rib and sternal fractures; pulmonary contusion
2	Female	39	Left	IV	2	Acromial and coracoid base fractures
3	Female	35	Right	V	3	-
4	Female	27	Right	IV	4	-
5	Male	55	Left	V	6	Multiple rib fractures, left pneumothorax, and scapular fracture
6	Female	37	Right	IV	2	Pelvic fracture, multiple rib fractures, and pneumothorax
7	Male	67	Left	V	3	Acromial fracture
8	Female	58	Left	IV	3	-
9	Male	57	Left	IV	5	Skull fracture, rib fracture
10	Male	53	Right	IV	4	-

**Table 2:** Constant score of patients

**Case**	**Preoperative**	**Postoperative**	**Outcome**
1	34	100	Excellent
2	18	83	Good
3	30	96	Excellent
4	26	94	Excellent
5	14	79	Good
6	25	96	Excellent
7	19	96	Excellent
8	32	96	Excellent
9	30	92	Excellent
10	24	92	Excellent
Mean	25.2	92.4	
Standard deviation	6.563	6.501	
*T* value	50.150		
*P* value	<0.001		

**Table 3:** **Visual analog scale** (**VAS**) **scores of patients**

**Case**	**Preoperative**	**Postoperative**
1	4	0
2	6	1
3	6	1
4	3	0
5	8	3
6	6	1
7	7	2
8	6	1
9	6	1
10	7	2
Mean	5.9	1.2
Standard deviation	1.449	0.919
*T* value	22.021	
*P* value	0.000	

## Discussion

The majority of acromioclavicular joint dislocations are caused by direct trauma. The force on the superolateral shoulder while falling causes inferior medial translation of the acromion relative to the distal clavicle. As the severity of the translation increases, the acromioclavicular ligament is first injured, followed by the coracoclavicular ligament, and finally, the deltoid and trapezius fascia [1]. The shoulder girdle, which is composed of the acromioclavicular, sternoclavicular, and scapulothoracic joints, plays an important role in maintaining the normal functions of the shoulder. Acromioclavicular joint dislocation not only produces such symptoms as pain and abnormal activity of the acromioclavicular joint, but also greatly affects the strength of the whole upper limb and flexibility of movement.

Biomechanical studies found that the horizontal stability of the acromioclavicular joint was mainly mediated by the acromioclavicular ligament. The superior and posterior acromioclavicular ligaments are the major structures responsible for limiting the posterior translation of the distal clavicle [10], producing 56% and 25% of the limitation, respectively [11], indicating that the acromioclavicular ligaments can effectively control the posterior translation of the distal clavicle. Although the inferior acromioclavicular ligament is thin, it is the main structure that limits the anterior translation of the distal clavicle [12]. Debski *et al*. found that transection of the acromioclavicular joint capsule resulted in a significant increase in anterior translation (6.4 mm) and posterior translation (3.6 mm) but not in superior translation [7]. Anterior and posterior loading leads to significantly increased forces in the coracoclavicular ligaments, suggesting that the coracoclavicular ligaments can partially compensate for the injured capsule in resisting horizontal loading conditions. However, the significant increases in the anterior translation and posterior translation demonstrate that the vertical coracoclavicular ligament cannot effectively restrain against anteroposterior instability. The main vertical stabilizer of the acromioclavicular joint is the coracoclavicular ligament, in which the conoid ligament served as a primary restraint against vertical loading, followed by the trapezoid ligament.

There are various surgical procedures for treating acromioclavicular joint dislocation, but none has yet become the golden standard procedure. Many surgical procedures are modified, but the same complications as the original methods are also present. Kirschner’s wire and tension band are commonly used at an early stage, and these exhibit advantages of easy performance and low cost. However, Kirschner’s wire may destroy the acromioclavicular joint surface and fibrocartilage plate, and is prone to induce acromioclavicular joint degeneration and traumatic arthritis. Considering that the acromioclavicular joint is a type of amphiarthrosis, rigid fixation via this joint leads to a high incidence of Kirschner’s wire breakage or loosening. A high incidence of complications is reported, and Kirschner’s wire may translate into the pleural cavity, spinal cord, and subclavicular space. The Bosworth screw method is a stabilization, with screws between the clavicle and the coracoid. Such a fixation impedes the synchronous rotation function of the clavicle and scapula [13], and leads to screw loosening or cut-out. This method is rarely applied currently. Stabilization of the coracoclavicular interval with a steel or titanium loop is a form of extra-acromioclavicular joint fixation, which does not aggravate the acromioclavicular joint degeneration, and enables movement in the acromioclavicular joint. However, the fixation has common complications of loop rupture, clavicle or coracoid fracture, and sub coracoid neurovascular injury. Moreover, loop stabilization is a non-anatomical reconstruction method, which leads to the anterior displacement of the distal clavicle in comparison with its normal anatomical location [14]. Since the clavicle hook plate was first used by Hachkenbruch *et al*. to treat acromioclavicular joint dislocation and good efficacy was achieved, it has been widely applied in clinical practices, and better results were achieved by surgical treatment with the hook plate than by conservative treatment although the coracoclavicular ligament was not reconstructed [15]. However, this method has problems of postoperative shoulder pain, acromial impingement, clavicle bone erosion, and fracture surrounding the fixator. The clavicle hook plate should be removed before the motion of the shoulder joint recovers to the point that the forearm extends to the vertex, namely about 8 to 12 weeks after surgery, while the premature removal of the plate may lead to the risk of loss of reduction [16]. The classical Weaver-Dunn procedure is to resect the distal clavicle, and transfer the coracoacromial ligament to reconstruct coracoclavicular ligament. Such a technique shows high efficacy in the treatment of chronic painful acromioclavicular joint dislocations [17]. Many biomechanical studies demonstrated weakness of the coracoacromial ligament after reconstruction, which was about 75% that of the intact coracoclavicular ligament [18,19]. It is reported that the postoperative failure of acromioclavicular joint reduction is 20%. In recent years, anatomical reconstruction of coracoclavicular ligaments with autologous tendon has been applied more and more widely [18,20,21]. Clinical and biomechanical studies showed that, compared with other methods, such a reconstruction obtained a strength and stiffness that was better approximated to the native structure, with obviously better clinical outcomes than that of the modified Weaver-Dunn procedure [22]. However, the reconstruction has the disadvantage of potential problems of complications on the donor sites [23]. In addition, the aforementioned methods only focus on coracoclavicular ligament reconstruction, without reference to acromioclavicular ligament reconstruction.

For acute acromioclavicular joint dislocation, particularly severe injuries of Rockwood type IV or greater, owing to ruptures of the acromioclavicular ligament, coracoclavicular ligament and delta-trapezius fascia, both static and dynamic stability of the acromioclavicular joint are destroyed; therefore, the acromioclavicular joint is unstable. The ideal treatment is to reconstruct each component of the acromioclavicular joint, that is, both the coracoclavicular ligament and the acromioclavicular ligament. This study presents a novel acromioclavicular ligament reconstruction technique designed by the authors, in combination with a double endobutton technique for coracoclavicular ligament reconstruction, to treat acute complete acromioclavicular dislocation. A double endobutton technique was used to restore the function of the conoid ligament anatomically, and its strength and stiffness exceeded the native ligament complex by approximately 40% [8,24], which provided good instant stability and enhanced the ligament’s endurance to cyclic loads. The weight of the upper limb and the loads produced by activities are distributed onto two button plates, which reduce the risk of clavicle or coracoid fractures. The plate of the endobutton is very thin, and this low-profile design reduces irritation of the surrounding tissues by the implants. The loop on the endobutton is a knotless design, which avoids soft tissue reactions caused by knotting. Such a method allows a certain motion in the acromioclavicular joint during abduction and elevation of the shoulder, which is in agreement with the physiological characteristic that the acromioclavicular joint belongs to amphiarthrosis [25]. Moreover, this method can be developed to provide an arthroscopic minimally invasive operation using suitable technology and equipment, and many studies report that arthroscopically endobutton reconstruction achieves satisfactory efficacy [25-27].

There are few studies reported in the literature regarding acromioclavicular joint reconstruction. Two previous studies selected the bone hole in the clavicle during coracoclavicular ligament reconstruction as the clavicular insertion site for acromioclavicular ligament reconstruction [19,28]. This bone hole is about 20 to 25 mm medially from the acromioclavicular joint, while the anatomical distance between the clavicular insertion of the normal acromioclavicular ligament and the acromioclavicular joint was 5.2 mm (women) and 7.6 mm (men) [29]. A longer distance between the clavicular insertion and acromial insertion of the ligament leads to poorer horizontal stability. In this study, the insertion site of the clavicle and acromion was reduced to within 8 mm, which was approximate to the anatomical position, to achieve better stability.

## Conclusions

This study used the double endobutton technique of coracoclavicular ligament reconstruction to restore vertical stability, and acromioclavicular ligament reconstruction with suture anchors to restore horizontal stability. Preliminary follow-up results for the ten patients indicated that the method significantly relieved pain, effectively improved the function of shoulder, which provided an effective surgical option for the patients, particularly those with acute complete acromioclavicular dislocation. Long-term follow-up and biomechanical testing of human specimens should be carried out to investigate the effect of this surgery on reduced incidence of long-term complications, such as acromioclavicular arthritis, and on whether the implanted reconstruction materials lead to instability due to relaxation and extension under cyclic loads.

### Consent

Written informed consent was obtained from the patient for the publication of this report and any accompanying images.

## Abbreviations

CT: Computerized tomography; PDS: Polydioxanone; SPSS: Statistical Package for Social Sciences; VAS: Visual analog scale.

## Competing interests

The authors declare that they have no competing interests.

## Authors’ contributions

HL designed the experiments. HL, CW, and JW did and interpreted the experiments. KW and DH wrote the manuscript. All authors read and approved the final manuscript.
